# Self-Injection Locking of a Vortex Spin Torque Oscillator by Delayed Feedback

**DOI:** 10.1038/srep26849

**Published:** 2016-05-31

**Authors:** Sumito Tsunegi, Eva Grimaldi, Romain Lebrun, Hitoshi Kubota, Alex S. Jenkins, Kay Yakushiji, Akio Fukushima, Paolo Bortolotti, Julie Grollier, Shinji Yuasa, Vincent Cros

**Affiliations:** 1 National Institute of Advanced Industrial Science and Technology (AIST), Spintronics Research Center, Tsukuba, Japan; 2 Unité Mixte de Physique, CNRS, Thales, Unive. Paris-Sud, Université Paris-Saclay, 91767 Palaiseau, France

## Abstract

The self-synchronization of spin torque oscillators is investigated experimentally by re-injecting its radiofrequency (rf) current after a certain delay time. We demonstrate that the integrated power and spectral linewidth are improved for optimal delays. Moreover by varying the phase difference between the emitted power and the re-injected one, we find a clear oscillatory dependence on the phase difference with a 2π periodicity of the frequency of the oscillator as well as its power and linewidth. Such periodical behavior within the self-injection regime is well described by the general model of nonlinear auto-oscillators including not only a delayed rf current but also all spin torque forces responsible for the self-synchronization. Our results reveal new approaches for controlling the non-autonomous dynamics of spin torque oscillators, a key issue for rf spintronics applications as well as for the development of neuro-inspired spin-torque oscillators based devices.

A major scientific breakthrough in spintronics was the introduction of spin transfer forces as a new means to generate high frequency nonlinear dynamics in nanoscale magnetic devices. The wealth of physics in spin transfer phenomena paves the way to a new generation of multi-functional spintronic devices^1^. Recent trends range from nanoscale radiofrequency (rf) devices for an efficient microwave source^2^ to highly sensitive microwave detection^3^,^4^, magnonic devices^5^ and more recently neuro-inspired memory devices^6^. For the purpose of realizing these applications, it becomes of paramount importance to not only identify and control the sources of noise^7^ but also to achieve a fine control of the phase of these spin torque devices^8^. Indeed, it is known and widely used in other types of oscillators such as conventional optical lasers^9^ or voltage control oscillators^10^, that the control of the oscillator phase can be achieved by a self-delayed feedback. In these systems, the spectral linewidth strongly depends on the delay time or phase difference between the oscillator and the re-injected signal, the effect of which can be observed in the forced synchronization of a spin torque oscillator (STO) with an rf current source. Here, the STO phase is determined by the phase of injected rf current^11^,^12^. V. Tiberkevich *et al.*^13^ proposed a similar implementation for an STO circuit based on the delayed self-injection of the output rf current. It should be noticed that the large nonlinear behavior, which is specific to STOs might detrimentally impact the self-locking process of the device^14^,^15^. However, more recently, Khalsa *et al.* reported in a theoretical study that the control of linewidth reduction could be expected in a STO circuit based on the delayed self-injection of the output rf current^16^. To our knowledge, this approach has not yet been addressed experimentally. We believe that the demonstration of the tuning of the rf properties through a controlled delay represents an important step for mastering the properties of STOs (frequency, spectral coherence and power consumption), which is crucial for the targeted rf applications^2^,^17^ as well for neuro inspired STO based memory devices^1^,^6^.

## Results and Discussion

Our main objective here is to identify the mechanisms of the self-injection locking of a vortex based STO using a delay line. In particular, we investigate the influence of the delay time Δ*t,* on the main rf characteristics of this new oscillating regime. The studied samples are composed of a circular FeB free layer in a magnetic tunnel junction (MTJ). The typical magneto-resistance (MR) ratio is about 120% at room temperature and the MTJ resistance is around 53 Ω at a bias voltage of 30 mV. For the FeB layer, the thickness and diameter were chosen so that the magnetic configuration at remanence corresponds to a magnetic vortex. All the measurements presented here have been carried out at room temperature with magnetic field of *H*_⊥_ = 3.0 kOe (the value necessary to have a large spin torque acting on the vortex^18^). However, similar results were obtained for other *H*_*⊥*_ values.

**Figure 1:**
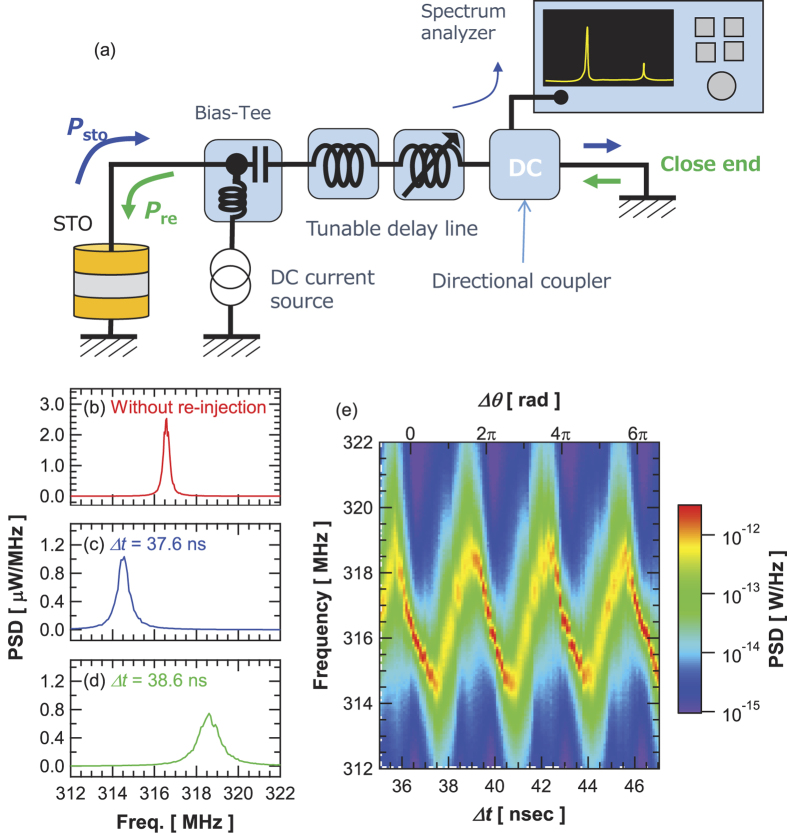
(**a**) Schematic of the delayed feedback circuit. Power Spectral Density (PSD) spectra at *I*_dc_ = 4.0 mA (**b**) without re-injection, with re-injection (**c**) Δ*t* = 37.6 ns, and (**d**) Δ*t* = 38.6 ns. (**e**) Color map of the PSD spectra as a function of delay time Δt at *I*_dc_ = 4.0 mA.

In Fig. 1b, we display a typical power spectral density (PSD) of the free-running STO i.e. without re-injection of the rf signal. The rf signal comes from the sustained vortex oscillations induced by the Slonczewski (or also called In-Plane) torque. This STO exhibits a frequency of 316.6 MHz, an integrated power of 1.1 μW and a full-width at half-maximum (FWHM) of 310 kHz recorded under *I*_dc_ = 4.0 mA. The amplitude of the dc current is about two times larger than the threshold current which is *I*_dc_ = 2.1 mA. In order to re-inject the rf signal generated by the STO into the oscillator, we use the measurement circuit described in Fig. 1a. The generated rf signal passes through a bias-tee, rf cables and eventually through the input port of a directional coupler. The close end at the output port of the directional coupler permits the reflection of the rf signal and injects the signal back into the STO with an intensity close to about 40% of the generated rf power. Note that most of the losses are from the cables. A tunable delay line is inserted in the circuit in order to precisely control the phase difference between the STO and the re-injected rf current which is defined as: Δ*θ* = 2π *f*_STO_ Δ*t* + π where *f*_STO_ is the STO frequency with re-injection and Δ*t* is the total delay time introduced by the circuit. This delay time Δ*t* comprises the delay due to the rf components and the delay due to the cables measured independently using a vector network analyzer (VNA). The last term π is added because of the phase shift which occurs at the close end. In the measurements presented here, the good impedance matching of the MTJ allows us to disregard the presence of stationary waves in this circuit (see Supplementary Materials). The coupled port of the directional coupler is used to measure with a spectrum analyzer (or a high frequency oscilloscope) the resulting rf signal generated from the STO after re-injection.

In Fig. 1c,d, we present PSD curves measured at *I*_dc_ = 4.0 mA when the rf signal is re-injected into the STO with two different delay times Δ*t* (obtained by adjusting the length of the tunable delay line). For Δ*t* = 37.6 ns (shown in Fig. 1c), we find that the frequency decreases down to 314.5 MHz i.e. 2.1 MHz lower than the free-running case. At the same time, the integrated power decreases to 1.02 μW. When the delay is tuned to Δ*t* = 38.6 ns (see Fig. 1d), the frequency becomes 318.6 MHz and the integrated power increases up to 1.18 μW which is the highest value that can be obtained by varying the delay time at *I*_dc_ = 4.0 mA. We also measure the PSDs obtained for longer delay time Δ*t* (in other words, a larger phase difference Δ*θ*). With these additional measurements (see Fig. 1e), we clearly observe a sinusoidal 2π−dependence of the STO peak frequency on delay time Δ*t*. To our knowledge, such oscillating dependence on Δ*θ* represents the first experimental demonstration of the self-injection locking of STO on its own rf emitted current.

**Figure 2:**
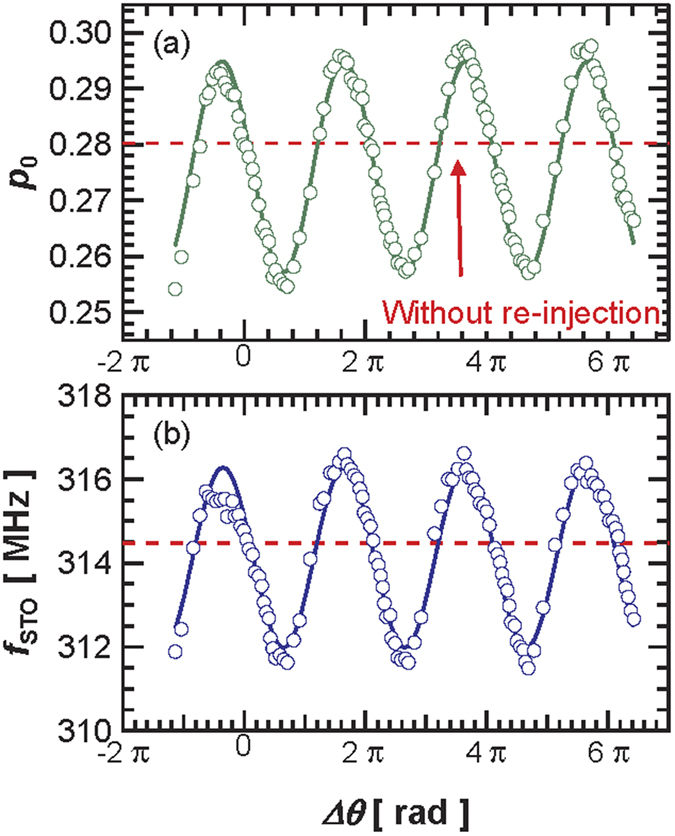
Measurements (**a**) the normalized power *p*_0_ and (**b**) the STO frequency *f*_STO_ evolution as a function of the phase difference Δ*θ* at *I*_dc_ = 3.7 mA. The dotted red lines are the values measured without re-injection (free-running STO) for the same external conditions. The solid lines are fitted by Eqs (1) and (2), respectively.

In the following, we focus on measurements of self-injection locking performed under the condition *I*_dc_ = 3.7 mA, at which the STO presents a relatively large nonlinear parameter *ν* of 4.1 as deduced from phase and amplitude noise analysis^19^,^20^. In Fig. 2a, we show again a clear 2π- dependence of the normalized power *p*_0_ (calculated from the square of the oscillation amplitude of vortex core) that varies between 0.255 and 0.295. As for the STO frequency *f*_STO_ with the phase difference Δ*θ* (see Fig. 2b), we find that its variation in the region between Δ*θ*  = 0 and Δ*θ* = 5π is around 0.8%, equivalent to 2.6 MHz of the value measured without re-injection (see dotted line in Fig. 2b). These experimental results clearly indicate that the re-injected rf current significantly modifies the limit cycle of the oscillating vortex core and defines a new oscillating regime. To quantify the amplitude of the rf re-injected current, we performed measurements using a VNA and found the amplitude to be about 80 μA i.e. about 2% of the dc current. We also stress that the self-synchronization has been achieved without any amplification of the rf current emitted by the STO.

To understand the main features of the mechanisms of self-injection locking of an STO using delayed feedback, we refer to the analytical study of this system recently done by Khalsa *et al.*^16^. Rewriting Eqs (5) and (8) of ref. 16 using the more conventional notations of the nonlinear auto-oscillator theory proposed by Slavin and Tiberkevich^21^ gives:


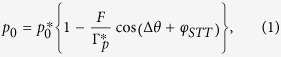



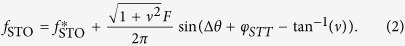


In these equations, 
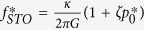
, 
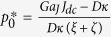
 and 
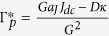
 are respectively the frequency, the normalized power and the relaxation damping rate of the stationary free-running STO. Several coefficients govern the dynamics of the oscillator: the vortex gyrovector *G*, the linear damping *D*, the nonlinear damping *ξ*, the linear confinement stiffness *κ*, the nonlinear confinement *ζ*. The Slonczewski torque efficiency aJ is associated with the perpendicular component of the spin polarization and responsible for the free-running spin transfer induced vortex oscillation^18^,^20^. The amplitudes of power and frequency variations depend on the strength of the normalized self-synchronization force *F*, expressed as 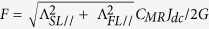 where *C*_*MR*_is a proportionality factor including the circuit losses and the MR ratio of the MTJ. *F* depends on the two spin torques capable of driving the vortex synchronization: the field like torque *Λ*_*FL*//_ and the Slonczweski torque *Λ*_*SL*//_ originating from the in-plane spin polarization. Both normalized power and frequency are expected to evolve as a sine function of the phase difference between the emitted and re-injected signal Δ*θ* = *θ*(*t*)−*θ*(*t*−Δ*t*). However, both are dephased with respect to each other when compared with Δ*θ*. This phase shift 
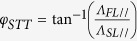
 depends on the relative magnitude of the two components of the spin torques. In addition, the frequency phase shift depends also on the nonlinearity factor of ν.

We now compare these analytical predictions to our experimental results. As shown in Fig. 2, both the normalized power *p*_*0*_ and frequency *f*_STO_ are well fitted by the predictions of Eqs (1) and (2), respectively. Equation (1) indicates that the power *p*_*0*_ should be inversely proportional to the relaxation damping rate 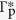. As detailed in the Supplementary Materials, we have been able to confirm this dependence with 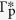, which further validates the self-injection locking model of Eq. (1). The changes of frequency *f*_STO_ should be directly linked to the non-linear parameter *ν* as expected from the prefactor of the sine in Eq. (2). In Fig. 2b, we find a frequency variation with Δ*θ* as large as 2.6 MHz when *ν = *4.1. For the measurements shown in Fig. 1e, a smaller variation amplitude (about 2.2 MHz) is obtained in agreement with a smaller *ν = *3.1 at *I*_dc_ = 4.0 mA. A more complete study can be found in the Supplementary Materials which also confirms the validity of the model. We now focus on the observed phase shifts of frequency and power. We first emphasize that in Fig. 2, *f*_STO_ and *p*_*0*_ are oscillating almost in phase, with only a very small phase difference of about 0.05π. This behavior is expected in highly non-linear oscillators. Indeed in Eq. (2) the term tan^−1^(*ν*) is always close to π/2 as long as the ν parameter is larger than 3 which is the case for all our measurements.

**Figure 3:**
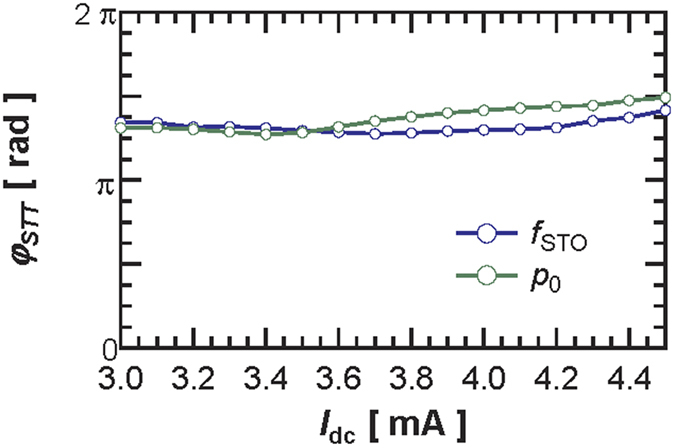
Evolution of the estimated phase shift φ_STT_ with the dc current *I*_dc_.

Using Eqs (1) and (2) and having evaluated the *ν* parameter, we can estimate the spin- transfer-forces phase shift *φ*_*STT*_ based on the dependence of *p*_0_ and *f*_STO_ with Δ*θ* (see Fig. 2a,b). Both dependencies result in a very similar *φ*_*STT*_ value, around 1.4π for *I*_dc_ = 3.7 mA. It should be noticed that a value close to 3π/2 as found in Fig. 3, implies that the field-like-torque drives the synchronization in our FeB MTJs i.e. *Λ*_*FL*//_ ≫ *Λ*_*SL*//_. This specific feature of vortex based STO is important as usually, the synchronization mechanisms equally depend on both *Λ*_*SL*//_ and *Λ*_*FL*//_ and on their signs. We have repeated the same analysis for different dc currents and have extracted the *φ*_*STT*_ dependence on *I*_dc_ (see Fig. 3). The evolution in the whole current range (between 3.0 and 4.5 mA) shows that *φ*_*STT*_ only increases slightly with *I*_dc_, presumably because of the different bias voltage dependences of the two torques^22^.

**Figure 4:**
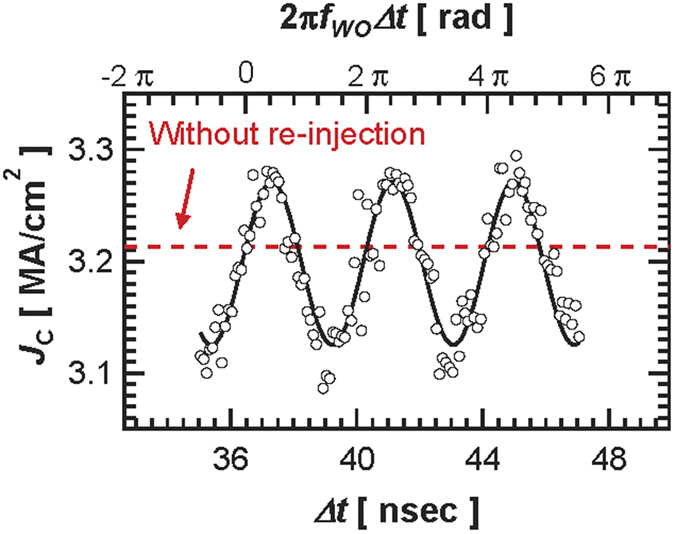
Critical current density *J*_C_ dependence on phase difference. The dotted red line is the value of the critical current density without re-injection (free-running STO). The solid line is fitted by Eq. (3).

Another important parameter of spin transfer induced oscillations is the threshold dc current *J*_*c*_ for sustained oscillations in the self-synchronized regime. In Fig. 4, we display the experimental threshold current *J*_c_ dependence on Δ*θ* that has been estimated from the inverse power *p*_0_ dependence on *J*_dc_ for different values of Δ*θ*. We find that *J*_c_ displays also a clear periodic behavior with Δ*θ* in agreement with Eq. (3). For particular values of the delay time, *J*_c_ is therefore decreased, which provides an interesting route to explore spin torque oscillators with reduced power consumption. This 2π-periodic evolution with Δ*θ* of the critical current *J*_*c*_ is also in agreement with the analytical model^16^:


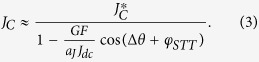


Here, 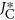 is the critical current of the free-running STO^18^. Note that in Fig. 4, the *φ*_*STT*_ values extracted from the analytical expression of *J*_*C*_ in Eq. (3) is again 1.6π, which is in excellent agreement with the one previously extracted from the *p*_0_ and *f*_STO_ evolutions shown in Fig. 3.

**Figure 5:**
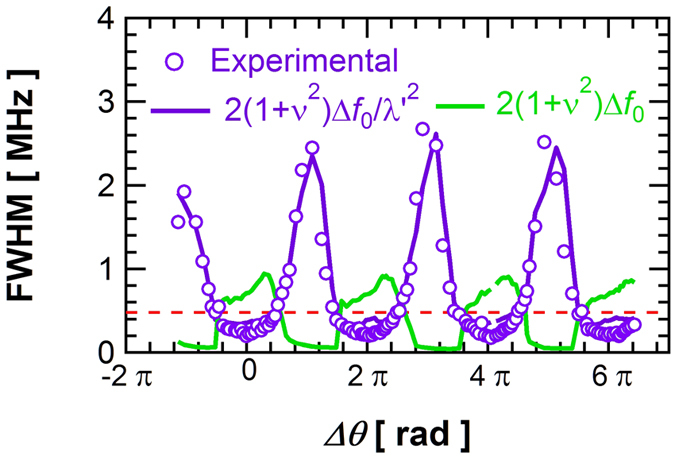
Evolution of the experimental spectral linewidth (opened purple circles) with the phase difference Δ*θ* at *I*_dc_ = 3.7 mA. The dotted red line is the value of the FWHM without re-injection (free-running STO). The solid purple curve corresponds to the predicted linewidth evolution obtained from Eq. (5) in the main text. The green curve describes the modification of the linewidth due only to a change of stationary regime.

We now focus on the impact of the self-injection process on the spectral quality of the STO. In Fig. 5, we display the evolution of the experimental linewidth (see purple dots) with Δ*θ* measured in the self-synchronized regime. Based on this mechanism related to the use of a tunable delay, we demonstrate that the STO linewidth can be reduced from 470 kHz in the free running regime down to 180 kHz in self-synchronized regime. This result clearly highlights the advantage of using a delay line from an application point of view, as it allows the optimization of the linewidth of the vortex STO via the phase shift Δ*θ* and delay time. In order to unravel the mechanisms responsible for the experimentally observed variation of the linewidth, we again compare our experimental results with analytical predictions. Khalsa *et al.* calculated that (for linewidth smaller than the typical relaxation rate Γ_p_), the linewidth of the self-synchronized regime can be expressed as:


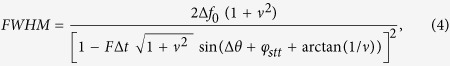


where 2Δ*f*_0_ is the linear linewidth associated with the self-synchronized stationary power *p*_0_.

By calculating the amplitude noise auto-correlation function of the self-synchronized STO (see Supplementary Material), we can extend this prediction and rewrite it in a more concise and physical manner as:


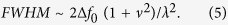


In this equation, λ is directly the factor renormalizing the relaxation damping rate in the self-synchronized regime: Γ_*p* self-sync = _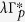. Analyzing the different terms in Eq. (5), we notice that the delay Δ*t* can influence the STO linewidth through two different mechanisms. The first mechanism is indirect. Indeed, the linear linewidth Δ*f*_0_ depends inversely on the power *p*_0_^20^,^21^, which oscillates with Δ*θ* as we have seen previously. If this mechanism is the main process for the linewidth evolution, then we expect to obtain linewidth maxima (resp. minima) for stationary power *p*_0_ minima (resp. maxima). In Fig. 5, we display the expected oscillating behavior of linewidth with Δ*θ* due to the change of the stationary regime i.e. only taking into account the numerator 2 Δ*f*_0_ (1 + ν^2^) (see green curve). We clearly see that the two curves show distinctly different behavior, thus discarding this mechanism of linewidth evolution with delay. The second mechanism which can lead to a change of linewidth is related to the factor λ renormalizing the relaxation damping rate and thus corresponds to the intrinsic noise filtering associated with the length of delay. In Fig. 5, we also plot the predicted evolution of 2Δ*f*_0_(1 + ν^2^)/λ ^2^ with Δ*θ* and a good qualitative agreement with the experimental results can be clearly seen, notably on the position of maxima and minima with Δ*θ*. This result shows that the measured large variation of linewidths induced by the delayed feedback is directly due to the modified phase and amplitude dynamics in the self-synchronized regime.

In conclusion, the self-synchronization of an STO has been successfully demonstrated for the first time by using a delayed feedback circuit. The self-synchronization induces new stationary regimes and endows the STO parameters with a periodic behavior. When the phase difference is appropriately tuned (by optimizing the delay time), we find that the STO spectral linewidth can be significantly reduced (more than 60% of reduction compared with the free running STO) and the emitted power increased compared with their respective values without self-synchronization. Such periodical behavior within the self-injection regime is well explained by considering the large field-like spin transfer force. This periodic behavior and enhancement of the spectral properties is not inherent to vortex oscillators, but should also be observable in other STO systems (i.e. non-vortex based STOs and nanocontacts). In light of the technological advantages obtained in our self-synchronized STOs which result from the precise control of the phase, a new avenue towards practical rf spintronics applications can be envisaged, as well as marking an important step towards the development of neuro-inspired STO based devices.

## Methods

The complete stack of the MTJ consists of buffer/PtMn(15)/Co_70_Fe_30_(2.5)/Ru(1.0)/Co_60_Fe_20_B_20_(2)/MgO(1.1)/Fe_80_B_20_(4.0)/MgO(1.1)/Ta(8)/Ru(7) where the subscript denotes the composition in atomic percent and the numbers in brackets indicate the layer thickness in nm (see ref. 23). Here, the top layer of the synthetic antiferromagnetic reference layer with uniform in-plane magnetization is the spin polarizing layer. The free layer made of FeB is covered with a MgO cap in order to decrease its magnetic damping that can be as small as 0.005^24^,^25^. After annealing at 360 °C in vacuum, magnetic tunnel junctions (MTJs) with radius of 150 nm were patterned by Ar ion milling and e–beam lithography. The component models of the measurement set-up and their manufacturers are listed in Table 1s in the supplementary information. The procedure for obtaining the auto-correlation function in the experiment is described in the supplementary information.

## Additional Information

**How to cite this article**: Tsunegi, S. *et al.* Self-Injection Locking of a Vortex Spin Torque Oscillator by Delayed Feedback. *Sci. Rep.* **6**, 26849; doi: 10.1038/srep26849 (2016).

## Supplementary Material

Supplementary Information
