# Supplementary material for: Self-Injection Locking of a Vortex Spin Torque Oscillator by Delayed Feedback
Source: Sci Rep. 2016 May 31;6:26849. doi: 10.1038/srep26849 (PMC4886513; doi:10.1038/srep26849)
Supplement: Supplementary Information [file srep26849-s1.pdf]

Supplementary Materials : Self-Injection Locking of a Vortex Spin Torque Oscillator by Delayed Feedback

S. Tsunegi[1,2], E. Grimaldi[1], R. Lebrun[1], H. Kubota[2], A.S. Jenkins[1], K. Yakushiji[2], A. Fukushima[2], P. Bortolotti[1], J. Grollier[1],S. Yuasa[2], and V. Cros[1]

[1]*Unité Mixte de Physique CNRS/Thales and Université Paris Sud, Palaiseau, France*
[2]*National Institute of Advanced Industrial Science and Technology (AIST), Spintronics Research Center, Tsukuba, Japan*

1) Manufacturer and model of the measurement set-up

Table 1s The rf component models in the delayed feedback circuits and their manufacturers.

	Manufacturer	Model	Performance
Bias-Tee	Keysight Technologies	11612A	Insertion loss : 0.1 dB
Delay line	Colby Instruments	PDL-100A-10ns	Frequency range : DC to 18 GHz<br>Resolution : 0.50 ps
Directional coupler	Mini-Circuits	ZFDC-10-182-S+	Frequency range : 10 to 1800MHz<br>Insertion loss : 1.5 dB<br>Coupling : 10 dB

2) Losses and total delay time in the measurement set-up

We experimentally estimate the delay time (Fig. S1a) and the loss in the circuit (Fig. S1b) for different delays generated by a tunable delay line. These measurements are carried out using a vector network analyzer (VNA) in the circuit shown in Fig. S1(c). The input rf power was -20 dBm. We observe that the range of delay is accessible with the tunable delay line from 0 to 6 ns. In the self-injection locking experiments, the actual delay range is doubled accounting for the double passes of the signal going in and out in the tunable delay line.

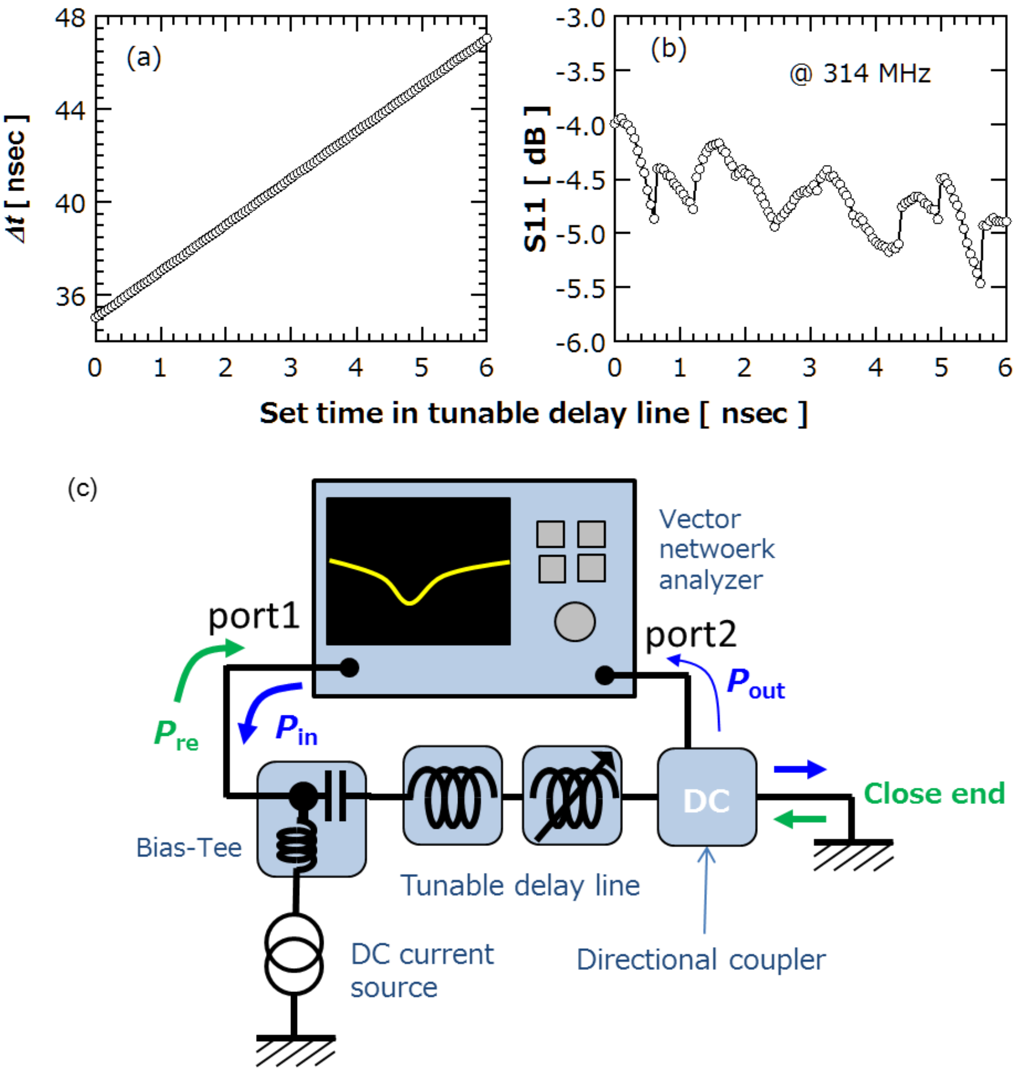

Fig. S1. (a) Total delay time and (b) loss of the circuit in self-injection scheme. (c) Outline of the circuit for measuring the delay time and loss for the self-injection.

3) Role of the impedance matching

When considering STO having large impedance mismatch, the rf signal is reflected at the STO. Fig S2 (a) shows the S11 parameter of three different STOs having resistance of 53, 23, and 17 Ω. These measurements have been performed using a circuit shown in Fig. S2(b) and with input power of -27 dBm. As expected, the reflection of rf signal is smaller for the STO having smaller impedance mismatch (close to 50 Ω). In consequence, in case of additional reflection taking place at STO, the resonator effect can be expected in the delayed feedback circuit due to two reflection points as we suggested in the main text.,

and thus the measured power also depends on the delay time or phase difference.

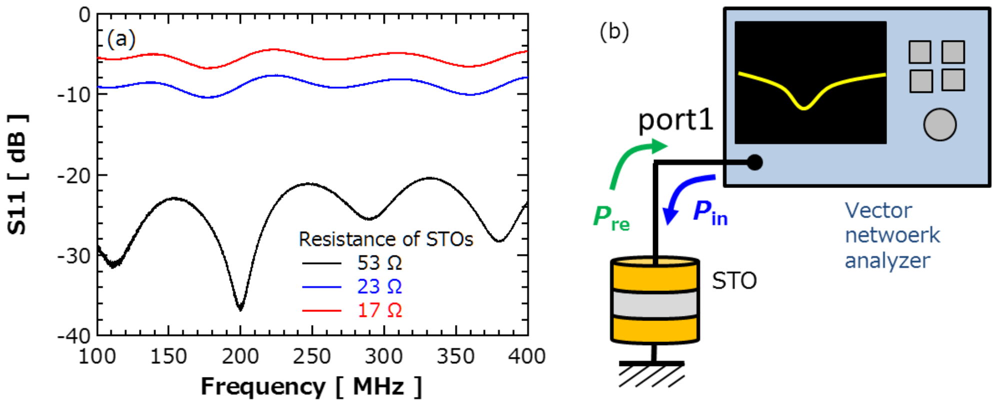

Fig. S2 (a) S11 parameter of several STOs having different resistances (b) Outline of S11 measurement.

4) Role of the relaxation rate Γ_p^* on p_0 variance

The variation of normalized power p_0 in self-synchronized regime is theoretically predicted to be inversely proportional to the relaxation damping rate of stationary free running STO, Γ_p^*. In Fig. S3, we show the experimental results of the Γ_p^* dependence of the p_0 variation amplitude. Here, the variation amplitude is described as $(p_0 - p_0^*)/ p_0^*$. It is clear that the variation is inversely proportional to the Γ_p^*.

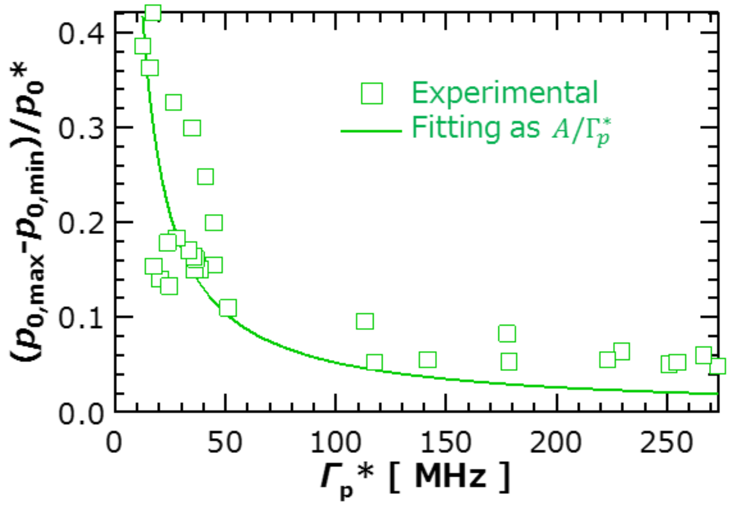

Fig. S3 The Γ_p^* dependence of the p_0 variation amplitude. The line was drawn using fitting parameter A

5) Role of the nonlinear parameter ν on the frequency variance

The variation of STO frequency in self-synchronized regime f_{STO} is theoretically predicted to be linked to the nonlinear parameter ν. In Fig. S4, we show the experimental

results of the ν dependence of f_{STO} variation amplitude. The variation of f_{STO} is described as $f_{STO,max}$- $f_{STO,min}$. The notation, $f_{STO,max(min)}$ indicates the maximum (minimum) value in the range of phase differences. The values of F estimated from the variation of p_0 and f_{STO} are 2.6 MHz and 4.1 MHz, respectively. This value is close to the theoretical value of 1.3 MHz, which is calculated from the amplitude of $\Lambda_{FL//}$ and $\Lambda_{SL//}$. This good agreement is also one of evidences that we demonstrated the self-synchronization by using delayed feedback circuit.

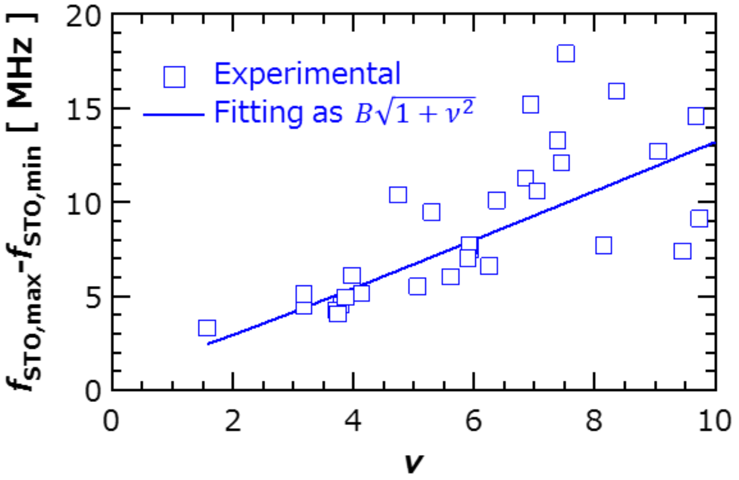

Fig. S4 : Dependence of ν *of the amplitude of variation of* f_{STO}.

6) Prediction of the spectral linewidth and amplitude auto-correlation function in the self-synchronized regime.

We can derive the expression of phase and amplitude noise power spectral density (PSD). For a white Gaussian noise, the amplitude noise PSD is obtained as:

$$S_{amp}(f) = \frac{\Delta f_0}{\beta\pi\left(\frac{f_p}{\beta}\right)^2} \frac{1}{\left(\frac{\beta f}{f_p}\right)^2 + \left[1 - F\Delta t\sqrt{1+\nu^2}\sin(\Delta\phi + \varphi_{stt} + \varphi_\nu)\right]^2}, \quad \text{(a.1)}$$

and the phase noise PSD is:

$$S_{phase}(f) = \frac{\Delta f_0}{\pi f^2} \frac{1 + \nu^2 + \left(\frac{f}{f_p}\right)^2 (1 + F^2\Delta t^2 \sin^2(\Delta\phi + \varphi_\nu))}{\left(\frac{\beta f}{f_p}\right)^2 + \left[1 - F\Delta t\sqrt{1+\nu^2}\sin(\Delta\phi + \varphi_{stt} + \varphi_\nu)\right]^2}, \quad \text{(a.2)}$$

with a the following factor β due to the delay

$$\beta = 1 + 2F\Delta t \cos(\Delta\phi + \varphi_{stt}) + F^2\Delta t^2,$$

The linear linewidth is expressed as :

$$\Delta f_0 = \frac{1}{4\pi}\Gamma_+(p_0)\frac{k_B T}{\varepsilon(p_0)} \ ,$$

where k_B is the Boltzmann constant, T is the absolute temperature and $\varepsilon(p_0)$ is the energy of the oscillator in the self-synchronized stationary regime.

For frequencies close to the frequency carrier i.e. $f << f_{\mathrm{p}}$, we obtain a random walk formula in the phase :

$$S_{phase}(f) = \frac{\Delta f_0}{\pi f^2}\frac{1+\nu^2}{\left[1 - F\Delta t\sqrt{1+\nu^2}\sin(\Delta\phi + \varphi_{stt} + \varphi_\nu)\right]^2}.$$

Thus, an the expression of the linewidth is deduced as

$$\mathrm{FWHM} = \frac{2\Delta f_0(1+\nu^2)}{\left[1 - F\Delta t\sqrt{1+\nu^2}\sin(\Delta\phi + \varphi_{stt} + \varphi_\nu)\right]^2}.$$

According to the Wiener Khintchine theorem, the auto-correlation function of the amplitude noise is related to the amplitude noise PSD by:

$$K_{amp}(t) = \int_{-\infty}^{+\infty} S_{amp}(f)e^{-i2\pi f t}df.$$

Thus, with Eq. (a.1) been given, we can estimate the auto-correlation function of the amplitude noise as:

$$K_{amp}(t) = \frac{\Delta f_0}{f_p\left[1 - F\Delta t\sqrt{1+\nu^2}\sin(\Delta\phi + \varphi_{stt} + \varphi_\nu)\right]}\mathrm{e}^{-2\pi f_p \frac{1 - F\Delta t\sqrt{1+\nu^2}\sin(\Delta\phi + \varphi_{stt} + \varphi_\nu)}{\beta}|t|}.$$

This can be rewritten as :

$$K_{amp}(t) = \frac{\pi\Delta f_0}{\Gamma_p\left[1 - F\Delta t\sqrt{1+\nu^2}\sin(\Delta\theta + \varphi_{stt} + \arctan(1/\nu))\right]}\ \mathrm{e}^{-2\Gamma_p\lambda|t|}, \qquad (6)$$

where Γ_p is the relaxation damping rate of the free running STO corresponding to the stationary power p_0^*, and λ is a renormalizing factor of Γ_p due to effects of the delay line mediated self-synchronization: $\lambda = \frac{1 - F\Delta t\sqrt{1+\nu^2}\sin(\Delta\theta + \varphi_{stt} + \arctan(1/\nu))}{1 + 2F\Delta t\cos(\Delta\theta + \varphi_{stt}) + (F\Delta t)^2}$

Importantly we highlight that, in our experimental conditions, the delay time and the re-injected current efficiency are relatively small ($F\Delta t$~ 0.03). Thus we can deduce Δf_0 and λ from the measured amplitude noise auto-correlation function and simplify the expression of the linewidth to FWHM $= 2\Delta f_0\ (1+\nu^2)/\lambda^2$.

7) How to obtain the auto-correlation function K_{amp} in experiment.

The time domain traces were recorded with a single-shot oscilloscope of 16 GHz electrical bandwidth. The oscilloscope we used was Keysight Technologies, model DSOX91604A. The duration time for the recorded trace was 3 ms with a sampling rate of 5 giga-samples per second (Gsam/s). Application of Hilbert transform method to the time domain traces was performed after the measurement, by which the phase and amplitude noise $\delta a(t)$ were extracted. Hilbert transform method has recently been proven to be successful in characterizing the noise in STO [19, 20 in main text]. Here, we used both a 400 MHz band pass filter centered at 315 MHz and a 32 MHz low pass filter to focus on the noise generated from STO. After that, we obtained the auto-correlation function from the calculation, $K_{\mathrm{amp}}(\tau) =< \delta a(t)\delta a(t-\tau) >$.
